# Correction: A green-lipped mussel reduces pain behavior and chondrocyte inflammation and attenuated experimental osteoarthritis progression

**DOI:** 10.1371/journal.pone.0321030

**Published:** 2025-03-25

**Authors:** JooYeon Jhun, Hyun Sik Na, Keun-Hyung Cho, Jiyoung Kim, Young-Mee Moon, Seung Yoon Lee, Jeong Su Lee, A. Ram Lee, Seok Jung Kim, Mi-La Cho, Sung-Hwan Park

The following information is missing from the Funding statement: This research is supported by a grant from the Korea Health Technology R&D Project, through the Korea Health Industry Development Institute (KHIDI), funded by the Ministry of Health & Welfare, Republic of Korea (grant number HI20C1496).
